# Mothers' and Caregivers' Knowledge and Experience of Neonatal Danger Signs: A Cross-Sectional Survey in Saudi Arabia

**DOI:** 10.1155/2019/1750240

**Published:** 2019-03-28

**Authors:** Amani Abu-Shaheen, Isamme AlFayyad, Muhammad Riaz, Abdullah Nofal, Abdulrahman AlMatary, Anas Khan, Humariya Heena

**Affiliations:** ^1^Research Center, King Fahad Medical City, Riyadh, Saudi Arabia; ^2^Epidemiology Department of Health Sciences, University of Leicester, Centre for Medicine, University Road, Leicester LE1 7RH, UK; ^3^King Saud University Medical City, Riyadh, Saudi Arabia; ^4^Neonatal Intensive Care Unit, King Fahad Medical City, Children Specialized Hospital, Riyadh, Saudi Arabia; ^5^Emergency Medicine Department, College of Medicine and University Medical City, King Saud University, Riyadh, Saudi Arabia

## Abstract

**Introduction:**

The majority of neonatal deaths in developing countries occur at home. Many of these deaths are related to late recognition of the signs of a serious illness by parents and a delay in the decision to seek medical care. Since the health-seeking behavior of mothers for neonatal care depends on the mothers' knowledge about WHO recognized danger signs, it is essential to investigate their knowledge of these signs.

**Objective:**

To investigate the knowledge and the experience of mothers and caregivers towards the WHO suggested neonatal danger signs.

**Methods:**

A community-based study was conducted on mothers who had delivered or had nursed a baby in the past two years.

**Results:**

A total of 1428 women were included in the analysis. Only 37% of the participant's knowledge covered three or more danger signs. The frequently reported participants' knowledge of danger signs in this study was for yellow soles (48.0%), not feeding since birth or stopping to feed (46.0%), and signs of local infection (37.0%). The majority (69.0%) of the participants had experienced at least one of the danger signs with their baby. The noteworthy frequent reports of the participants' experiences were for yellow soles (27.0%), not feeding since birth or stopping to feed (25.0%), and umbilical complications (19.0%).

**Conclusion:**

The proportion of mothers with knowledge of at least three neonatal danger signs is low. There is a need for developing interventions to increase a mother's knowledge of newborns danger signs.

## 1. Introduction

According to the World Health Organization (WHO), the early newborn period is the most critical for survival for a neonate [1]. In 2015, 5.9 million children died worldwide before their fifth birthday [2]. These deaths are mostly from preventable causes and occur mainly in developing countries [3]. Approximately, 45% of all under-five child deaths are among newborns in their neonatal period. Three-quarters of newborn deaths occur in the first week of life with 25 to 45% in the first 24 hours of life [4].

The majority of neonatal deaths in developing countries occur at home, up to two-thirds of which can be prevented if timely and efficient health measures are taken [5]. A lot of these deaths are due to late recognition of the signs of a serious illness by parents and caregivers and a delay in the decision to seek medical care at the onset [6–8].

Preventing mortalities by enhancing the child health in the community is at the principal of the approach named Integrated Management of Childhood Illness (IMCI), which was developed by UNICEF and the WHO in 1992 to prevent or detect and treat the top childhood killers [9]. The IMCI initiative adopts a cross-cutting approach recognizing that, in many cases, more than one underlying cause can lead to the child illness. IMCI attempts to combine the lessons learned from various preventive nationwide programs into an effective approach for managing the sick child. IMCI attempts to decrease childhood mortality and morbidity by enhancing family and community practices for the home management of illness [10].

Immediate recognition of the danger signs mentioned in IMCI is the first and most important clue a mother or a caregiver could perceive to seek medical attention. The danger signs recognized by WHO could indicate a severe disease or a local infection. The danger signs for a severe disease are refusal of feeds, convulsions, fast breathing, fever, low body temperature, severe chest in-drawing, and movement only when stimulated or no movement at all. The signs of local infection are umbilical redness or draining pus and skin pustules [10].

In Saudi Arabia infant mortality rate is 13 deaths per 1000 live births in 2015 [5]. Although under-five mortality rate in Saudi Arabia has reached the Millennium Developmental Goal-4 target (two-thirds reduction in neonatal mortality rate by 2015), infant mortality rate is still higher than most of Gulf Cooperation Council (GCC) and developed countries [5]. As the mothers' health-seeking behavior for neonatal care depends highly on their knowledge about WHO recognized danger signs, it is important to investigate the knowledge of these signs, which has been hardly investigated in Saudi Arabia. Thus, this study aims to investigate the knowledge of mothers and caregivers of the WHO suggested newborn danger signs in Riyadh, Saudi Arabia.

## 2. Material and Methods

### 2.1. Study Design and Sampling Technique

A community-based cross-sectional study was conducted in Riyadh City of Saudi Arabia to investigate the knowledge of Saudi mothers and caregivers towards the WHO neonate's danger signs. In 2013, the literacy rate, among adult females (% of females 15 and above), is 91.37 [11]. The fertility rate was 2.6 in 2013 [12] and the infant mortality rate per 1000 live births in Saudi Arabia was 12.50 in 2015 [13]. Riyadh, the capital city of the Kingdom of Saudi Arabia, is also the largest city in the kingdom and home to over 8 million people (2017) [14]. Riyadh is divided into five districts and each district in turn contains several primary health care centers (PHCCs) affiliated to the Ministry of Health (MOH). PHCCs are located in most localities of the city and provide free of charge primary medical care services for the majority of the residents without referrals. Sampling was performed in two stages; after obtaining a list of all PHCCs from the department of census in MOH, random samples (10%) of the PHCCs in each district were selected in stage one, and then mothers or caregivers were approached and invited to participate in this study from the waiting room of the selected PHCCs in stage two in sample of proportionate sizes.

### 2.2. Study Subjects

All mothers who delivered during the past two years or have nursed a baby in the past two years (in case of caregivers) were considered in the sampling process. Caregivers included grandmothers, grandfathers, fathers, or “nannies” (other female relatives). Women that were unable to provide information during the data collection period were excluded.

### 2.3. Data Collection

Face to face interviews were conducted with the mothers at the PHCCs by trained research assistants using a structured questionnaire that was piloted for ambiguity before the study began. The interview was designed after an in-depth literature review [15–17].

The information collected included the sociodemographic characteristics of the neonate and the mothers/caregivers including age, gender of the neonate, area of residence, education level, monthly income, occupation, and number of children or number of children nursed by caregivers. Information on the reproductive history of pregnant women and mother's knowledge, experience, and response for the neonate's danger signs was also collected. Participants were asked to list the signs they would consider to be serious health problems and might threaten the neonate's life. They were also asked to list any of the signs that they experienced personally with their neonate, the actions they took, the help they sought from the healthcare institution, and reasons for not utilizing the services of any healthcare facility. They were requested to recall the time from noticing the danger sign(s) and presentation to the health care facility, promptness of care received in the health facility, and outcome of their neonate's illness. The signs were clustered according to the Nine [9] signs identified by the WHO. The danger signs were based on the WHO definition and described as follows: not feeding since birth or stopped feeding, convulsions, fast breathing (respiratory rate of 60 or more), severe chest indrawing (difficulty in breathing), fever (temperature of ≥ 37.5°C), hypothermia (temperature ≤ 35.5°C), weakness or lethargy (only moves when stimulated or not even when stimulated), yellow soles (sign of jaundice), and sign of local infection (umbilicus redness or draining pus, skin boils, or eyes draining pus).

### 2.4. Ethical Considerations

Informed consent was obtained before each participant's enrollment, the identity of the participant was kept confidential, and the institutional review board approval was obtained from the Directorate of PHCCs, Ministry of Health, Riyadh, and from King Fahad Medical City, Riyadh, Saudi Arabia.

### 2.5. Sample Size Estimate

Based on the infant mortality and fertility rates, the estimated population of neonates at risk is 200,000 across Saudi Arabia. Estimated sample size of (N = 1425) for the study was calculated with the assumptions of precision = 1.00 %, prevalence = 1.25 %, population size = 200,000, and 95% Confidence interval specified limits of 0.25%-2.25% (these limits equal prevalence plus or minus precision).

### 2.6. Statistical Analysis

Data analysis was done using Stata (version 12) software. Percentages and frequencies were used to describe the sociodemographic characteristics. Frequencies and percentages (95% Confidence Intervals-CI) were computed for the mother's knowledge and experience of neonate danger signs (9 items). A total score (total number of correct spontaneous answers to nine items with a minimum score of zero and maximum of nine) was computed to measure the mother's knowledge about the danger signs and a mother's knowledge was considered to be satisfactory if she had the knowledge of at least 3 danger signs (i.e., a score of 3 or above) [18].

## 3. Results

The sociodemographic characteristics of 1428 women included in the analysis are described in Table 1. Among them 73.0% were Saudi nationals and they had delivered in the past 1-24 months. They were selected from 5 regions of Riyadh city (central 16.7%, West 16.7%, East 36.5%, South 13.4%, and North 16.7%). The majority of women (70.0%) were in the age range of 26-40 years (range: 18-50). Slightly more than half (52.0%) of their neonates were female, and the majority (76.0%) of those neonates were in the age group of [1–10, 19, 20] months old (overall range: 1-24 months) at the time of conduct. Almost all the neonates (98.0%) were cared for by their mothers. Thirty-three percent of the mothers were educated to a degree level and 37.0% had secondary education. The majority of the women (83.0%) were unemployed and 50.0% had a very low income. Around 45.0% had 4 or more children and 46% did not have any experience with abortion. Above three fourths (87.0%) of the women had attended the recommended visits of antenatal care (>4 visits).

### 3.1. Participants' Knowledge of Neonate's Danger Signs

Participants' knowledge of neonate's danger signs is summarized in Table 2. The majority (89.0%) of the participants were knowledgeable about at least one of the danger signs, but only 37.0% (95% CI: 34.9-40.0) had knowledge of three or more danger signs (satisfactory knowledge). In this study, the frequent reports of participants' knowledge of danger signs were for jaundice 48.0% (95% CI: 45.6-50.8), not feeding since birth or stopped feeding 46.0% (95% CI: 43.42-48.5), sign of local infection 37.0% (95% CI: 34.6-39.6), fever 31.0% (95% CI: 28.8-33.6), and convulsion, 18.0% (95% CI: 16.3-20.3). The other signs, listed in Table 2, were reported in less than 15.0% of the participants.

### 3.2. Participants' Experience of Neonate's Danger Signs

The majority 68.8% (95% CI: 66.3-71.2) of the participants had experienced at least one of the danger signs with their baby. In this study, the frequently reported danger signs were sign of jaundice 27.1% (95% CI: 24.8-29.4), not feeding since birth or stopped feeding 24.7% (95% CI: 22.4-26.9), sign of local infection 19.3% (95% CI: 17.2-21.3), and fever 18.6% (95% CI: 16.6-20.6). Experiences of the other danger signs, listed in Table 3, were reported in less than 10% of the participants.

About 635 (44.5) of the mothers/caregivers sought medical care, of whom 285 (46.9%) sought private hospital and 217 (35.7%) sought PHCCs. The time lapse between occurrence of the neonatal danger signs and seeking the help of the medical care was (10.4±12.1 hours). Out of the 592 of the mothers/caregivers who reported the final outcome of their neonates' experience with danger signs, one baby death was reported and 9 babies developed complications (Table 4).

## 4. Discussion

Adequate mother's and/or caregiver's knowledge of neonate danger signs is important for reducing infant mortality and morbidity. In this study, we assessed mother's knowledge of the key danger signs of infants. Slightly more than one-third of the women appeared to have a satisfactory knowledge of the neonate danger signs (knowledge of at least three signs) and the proportion of women with knowledge of each frequently reported danger sign was even less than fifty percent. The majority reported that they have had an experience of at least one danger sign with their baby, which is corroborated with the proportion of women that appeared to know at least one danger sign. Previous studies in different setting have revealed varied differences in women's knowledge of neonatal danger signs. The proportion of women knowing at least one danger sign in this study is congruent with several studies [15, 16, 21, 22]. Abdulrida et al. (2015) assessed the knowledge and health-seeking practices of mothers attending (n=275) PHCCs in Bagdad (Iraq) about danger signs in neonates. Abdulrida reported that all of the study participants' mothers mentioned correctly at least one danger sign [22]. Similarly, Salem et al. (2018) reported a high proportion of knowledge of one danger sign among women (n= 372) living in Ambanja, Madagascar (Salem 2018) [21]. In contrast to our findings, other studies conducted in Ethiopia, Uganda, and Nigeria have reported low proportions of mothers' knowledge of at least one danger sign [17, 23–25].

In present study, the proportion of mothers who reported at least three danger signs was low (37%), which was lower than the proportion reported (81%) in Iraq by Abdulrida et al. (2015) [22]. Despite this, our findings were better than those of the proportions (almost two times) reported from Ethiopia (18.2%) by Nigatu et al. [21] and from Kenya (15.5%) by Kibaru and Otara [26], but the proportion of women with knowledge of at least three danger signs is higher than that reported by others [25, 27, 28].

In this study, a low level of mothers' knowledge of the neonate's danger signs was observed even though the majority of the women had attended the recommended > 4 visits of antenatal care; this led to an idea that the antenatal care providers may not have proper resources and facilities to educate mothers about the neonate danger signs. It is also possible that the low level of mothers' knowledge could be contributed to their socioeconomic circumstances such as lack of higher educational achievement, low income, and access to social activities. Previous studies in different settings have identified factors that are significantly associated with mothers' knowledge of neonate's danger signs [21, 22, 26, 27]. For example, a regional study conducted in Iraq indicated that mothers' level of education, employment, and attendance of higher number of antenatal care visits are associated with improved mothers' knowledge of neonatal danger signs [22]. Moreover, Nigatu from Northwest of Ethiopia has reported mother's good knowledge of danger signs, mother's and father's higher education, attending antenatal and postnatal cares, and having access to television [21]. Another community based study as well from Southern Ethiopia reported that area of residence and knowledge about essential neonate care were associated significantly with the mother's knowledge of neonatal danger signs [29].

Mothers with sound economic status are expected to have access to better health care services and other resources such as exposure to media especially television to learn about the neonate's health [23].

In our study, less than half of the mothers/caregivers reported seeking medical facilities for danger signs management. In study done in Iraq, it was revealed that 25.4% of the mothers sought medical facilities for danger signs management [22].

In study conducted in Pakistan, it was showed that 69.4% had sought private sector and 11.7% sought government sector [30]. Moreover, another study was conducted in Iraq, which had revealed that 55.2% of the mothers sought governmental hospital and 17.9% had sought private hospitals [22]. However, the current study found that 46.9% of the mothers had sought private hospitals and 17.4% sought care from government hospitals. These differences in seeking different medical facilities among countries could be contributed to the quality of services provided by these facilities. In a study conducted in Iraq [22], it was showed that 61.2% of the mothers presented their neonates to the medical facilities within less than 24 hours after recognition of the danger signs and about one-quarter after more than 24 hours. In contrast, the mothers in our study reported better prompt behavior in seeking medical help within 10 hours.

### 4.1. Strengths and Limitations

Parts of the strengths of this study include the high number of participating women and being conducted in community-based settings across the five regions of Riyadh, the capital of Saudi Arabia. The study was rigorously conducted using multistage sampling techniques, recruiting a large sample of women, and including women from a wider geographical area in Riyadh. The questionnaires were completed by a research assistant in a face to face interview of the mother or care provider at PHCCs, which ensured the accuracy of the information collected. As reported in a study of clinical signs of younger infants [18], we used a similar cut-off of the mother's knowledge of at least three danger signs to be a satisfactory knowledge. However, there are some limitations including the following: fifty-eight percent of the babies in this study were older than six months and a relatively long period may have induced a recall bias in the mother's true knowledge of neonate's danger signs. Therefore, the results of this study need to take account of these limitations for generalizability and citations.

## 5. Conclusion

Although the participants' experience of the neonatal danger signs is high, the proportion of mothers/caregivers knowing at least three danger signs is still low in this community-based study. Therefore, in Saudi Arabia, the public health and educational policymakers are required to consider developing interventions strategies for increasing mother's knowledge and awareness of neonatal danger signs to reduce infants' mortality and morbidity. Such strategies should focus on training of health care workers and establishing a rigorous supportive supervision for quality assurance and sustained health education by utilizing maternal child health booklets as interventions modalities followed by continuous evaluation to confirm the validity of these interventions.

## Figures and Tables

**Table 1:** Participants' characteristics (N=1428).

Variables	Frequency (%)
*Participants/Mother's Age (in years)*	
18-25	327 (22.9)
26-30	411 (28.8)
31-40	587 (41.1)
40-50	102 (7.2)
*Neonate's age (in months)∗*	
1-6	596 (42.1)
7-12	489 (34.5)
13-18	145 (10.2)
19-24	187 (13.2)
*Neonate relationship with the participant*	
Mother	1397 (97.8)
Health caregiver	31 (2.2)
*Neonate's gender (Female)∗*	727 (51.7)
*Area of residence (inside Riyadh)*	1298 (90.9)
*Riyadh region*	
Central	239 (16.7)
West	238 (16.7)
East	521 (36.5)
South	192 (13.4)
North	238 (16.7)
*Level of education*	
Illiterate	152 (10.6)
Primary school	87 (6.1)
Middle School	188 (13.2)
Secondary School	533 (37.3)
Bachelor	430 (30.1)
Higher Education	38 (2.7)
*Participants' monthly income (Saudi Riyals)∗*	
0-5000	557 (49.6)
6000-10000	432 (38.5)
10000 and above	134 (11.9)
*Occupation*	
Government	170 (11.9)
Private Sector	66 (4.6)
Unemployed	1192 (83.5)
*Number of children*	
1-3	881 (62.4)
4-5	387 (27.4)
≥6	145 (10.3)

*∗* Data is missing in participants' income for n=305, in neonate's gender for 21, and in neonate's age for 10.

**Table 2:** Participants' knowledge (recognition) of the neonatal danger signs.

Danger Signs	Frequency (%)	95% CI
Not feeding since birth or stopped feeding	657 (46.0)	43.4-48.5
Convulsion	261 (18.2)	16.3-20.3
Fast breathing	156 (10.9)	9.3-12.5
Severe chest indrawing	137 (9.6)	8.1-11.1
Fever ≥ 37.5°C	445 (31.2)	28.8-33.6
Hypothermia ≤ 35.5°C	71 (5.0)	3.8-6.1
Weakness or lethargy	171 (12.0)	10.3-13.7
Yellow soles	688 (48.2)	45.6-50.8
Sign of local infection	530 (37.1)	34.6-39.6
knowledge of at least 3 of the above danger signs	535 (37.5)	34.9-40.0

**Table 3:** Participants' experience of the neonatal danger signs.

Danger Signs	Frequency (%)	95% CI
Not feeding since birth or stopped feeding	352 (24.7)	22.4-26.9
Convulsion	93 (6.5)	5.2-7.8
Fast breathing	54 (3.8)	2.8-4.8
Severe chest indrawing	59 (4.1)	3.1-5.2
Fever ≥ 37.5°C	266 (18.6)	16.6-20.6
Hypothermia ≤ 35.5°C	15 (1.1)	0.5-1.6
Weakness or lethargy	59 (4.1)	3.1-5.2
Yellow soles	387 (27.1)	24.8-29.4
Sign of local infection	275 (19.3)	17.2-21.3
Experience of at least one of the above danger signs	982 (68.8)	66.3-71.2

**Table 4:** Seeking the medical care subsequent to experience of neonatal danger signs.

*Sought medical care *	635 (44.5)
*Treatment was received in *	
Private hospital	285 (46.9)
PHCCs	217 (35.7)
Governmental Hospital	106 (17.4)
*Mean time to seeking the help of the medical care (hours) *	10.4 (±12.1)
*Final resolution of the outcome*	
Resolved	582 (98.3)
Complicated	9 (1.5)
Died	1 (0.2)

Data presented as number (%) and mean (±SD).

## Data Availability

The data used to support the findings of this study are available from the corresponding author upon request.
